# The c-di-GMP effector FleQ controls alginate production by repressing transcription of algD in Azotobacter vinelandii

**DOI:** 10.1099/mic.0.001556

**Published:** 2025-04-24

**Authors:** Víctor V. Barrios-Rafael, Carlos L. Ahumada-Manuel, Scherezada Orgaz-Ramírez, Jessica Nava-Galeana, Josefina Guzmán, Soledad Moreno, Víctor H. Bustamante, Cinthia Núñez

**Affiliations:** 1 Departamento de Microbiología Molecular, Instituto de Biotecnología, Universidad Nacional Autónoma de México (UNAM), Cuernavaca, Morelos, México

**Keywords:** *Azotobacter vinelandii*, alginate, *algD*, c-di-GMP, FleQ, encystment

## Abstract

Production of the exopolysaccharide alginate by *Azotobacter vinelandii*, member of the *Pseudomonadaceae* family, is positively controlled by the second messenger c-di-GMP. This effect was solely attributed to the role of c-di-GMP in activating the alginate polymerase complex. In this study, the role of c-di-GMP in *algD* transcription, which encodes the key enzyme for alginate synthesis, was investigated. *algD* transcription correlated with artificially high or low levels of c-di-GMP. Moreover, FleQ, one of the best-characterized c-di-GMP effectors, was found to exert a negative effect on alginate production and *algD* transcription, as both increased in a Δ*fleQ* mutant relative to the wild-type strain or the Δ*fleQ*/*fleQ*+ complemented strain. Electrophoretic mobility shift assays (EMSAs) confirmed that FleQ directly binds to the regulatory region of *algD*, which was consistent with the presence of two FleQ binding sites in the vicinity of the *algD* RpoS-dependent promoter. In *A. vinelandii*, c-di-GMP is essential for the expression of alginate C-5 epimerases (AlgE1-6), which are necessary for structuring the envelope of differentiated cells, known as cysts. However, FleQ was not involved in this regulation. Collectively, our results support a model in which *algD* transcription is under the positive control of c-di-GMP, while FleQ may only partially mediate this effect. In contrast, our study revealed a FleQ-independent regulatory mechanism for the control of *A. vinelandii* encystment.

## Graphical Abstract

In addition to activating the alginate polymerase complex, we found that the second messenger c-di-GMP is necessary for the transcriptional activation of the *Azotobacter vinelandii algD* gene, which encodes the key enzyme for alginate biosynthesis. FleQ was identified as a transcriptional regulator that exerts a repressive effect by directly binding to the *algD* regulatory region. Moreover, c-di-GMP was found to be essential for the expression of genes encoding alginate modification enzymes (AlgE1-6), which are involved in cyst capsule formation. However, our data indicate that FleQ is not involved in this regulation.

**Impact Statement**

This work documents the positive control of *algD* transcription by the second messenger c-di-GMP in *Azotobacter vinelandii*. Notably, FleQ was identified as a direct repressor of *algD* transcription, a function that has not been previously described in *Pseudomonas* spp. This study expands the known role of the second messenger c-di-GMP and the transcriptional regulator FleQ in the control of bacterial exopolysaccharide production.

## Introduction

*Azotobacter vinelandii,* belonging to the *Pseudomonadaceae* family, is a free-living, nitrogen-fixing bacterium that is motile due to the presence of peritrichous flagella. The *Azotobacter* genus undergoes a differentiation process to form desiccation-resistant cysts [1]. During encystment, biochemical, metabolic and morphological changes occur, including the loss of flagella and the arrest of nitrogen fixation, among many others. The central body of the mature cyst is surrounded by two protective layers, the exine and the intine, which contain a high proportion of the exopolysaccharide alginate, essential for dehydration resistance [2]. Given their nitrogen-fixing capabilities and plant growth-promoting traits, *Azotobacter* species have great potential as biofertilizers, enhancing plant nutrition and soil fertility [3]. *Azotobacter*-based biofertilizers stand out due to these bacteria’s ability to form cysts, which confer resistance to environmental stresses and ensure stability under diverse conditions, potentially contributing to sustainable agriculture [3].

Alginate is a linear polysaccharide composed of two monomers: α-l-mannuronate (M) and its C-5 epimer, β-d-guluronate (G). Alginate synthesis begins with fructose-6-P, which, after three enzymatic steps in the cytosol, is converted into the activated precursor GDP-mannuronate. This precursor serves as the substrate for the alginate polymerase complex, located in the inner membrane [4,6]. The product of the *algD* gene catalyses the oxidation of GDP-mannose to GDP-mannuronate. This reaction is the rate-limiting step of the pathway; hence, *algD* expression is subjected to complex genetic regulation [4]. Transcription of *algD* is initiated from three promoters: the P1 promoter, dependent on RpoS; the P2 promoter, dependent on AlgU; and the P3 promoter, controlled by an unknown sigma factor [7,10]. In contrast to *P. aeruginosa*, where several transcriptional regulators, such as AmrZ, AlgB or AlgR, directly regulate *algD* [11,14], no direct transcriptional regulators have been reported for *A. vinelandii algD* so far [4].

Previous work revealed that the second messenger bis (3′,5′)-cyclic dimeric guanosine monophosphate (c-di-GMP) exerts a positive effect on the amount of alginate produced by *A. vinelandii* and on its molecular mass [15,16]. The second messenger c-di-GMP controls a vast array of cellular processes in bacteria, including the transitions from the motile to the sessile style, biofilm formation or the progression of the cell cycle [17]. It is produced from GTP by the activity of diguanylate cyclases (DGC) and degraded by phosphodiesterases (PDE). c-di-GMP effectors convert changes in c-di-GMP concentration into cellular responses and such effectors include transcription factors, riboswitches or signalling proteins [18]. Under the vegetative growth of *A. vinelandii*, a c-di-GMP module composed of the DGC *Av*GReg and the PDE MucG provides the c-di-GMP necessary for alginate production [15]. The absence of MucG increases c-di-GMP levels and the production of alginate with higher molecular mass, when compared to the wild-type (wt) strain [15,19]. Since the alginate polymerase complex, Alg8-44, is activated by binding of c-di-GMP to the co-polymerase Alg44 [20,21], these alginate phenotypes were attributed to increased activation of Alg8-44. However, higher *algD* expression was observed in the absence of the PDE MucG [19], suggesting that c-di-GMP affects *algD* transcription through an unknown mechanism.

In *A. vinelandii*, c-di-GMP was also found to be essential for the formation of mature cysts [16]. The alginate protective layers of the cysts, essential for desiccation resistance, contain different proportions and distributions of G residues which result from the activity of a family of C-5 epimerases, encoded by the genes *algE1-6*. The second messenger c-di-GMP was necessary for expression of *algE1-6* genes and, therefore, to produce mature cysts resistant to desiccation [16].

FleQ is one of the best c-di-GMP effectors so far characterized in *Pseudomonas* species [22]. Although FleQ shares significant homology with members of the NtrC family, containing an N-terminal receiver domain (REC), a central ATPase (AAA+) domain and a C-terminal HTH DNA binding motif [23], its activity responds to changes in the levels of c-di-GMP altering target promoter activity and it is also influenced by the FleN antagonist and ATP hydrolysis [24,26]. In *P. aeruginosa,* FleQ is the master regulator of flagellar motility and exopolysaccharide production, mediating the transition from the planktonic to the biofilm lifestyles. At low c-di-GMP, FleQ activates flagellar genes but represses genes encoding biofilm matrix components, such as the Pel polysaccharide [22]. However, at high c-di-GMP, FleQ is unable to activate the flagellar genes but upregulates the expression of genes encoding matrix components necessary for biofilm formation [22].

In this study, the role of *A. vinelandii* FleQ as a possible intermediary in the regulation of *algD* transcription and mature cyst formation by c-di-GMP was investigated. We found that FleQ represses *algD* transcription and only partially mediates the positive control of this gene by c-di-GMP. In contrast, our results revealed that FleQ is not involved in regulating the genes encoding alginate C-5 epimerases and is not essential to produce mature cysts resistant to desiccation.

## Methods

### Strains and cultivation conditions

The wt strain AEIV of *A. vinelandii* was used [27] and routinely cultivated in Burk’s medium with sucrose (20 g l^−1^) as the carbon source (Burk’s sucrose medium). The composition of the growth medium has been described previously [28]. Cultures were incubated at 30 °C and 200 r.p.m.

Resistance to desiccation of *A. vinelandii* cysts was determined as reported previously [7,16]. Transformation of *A. vinelandii* was carried out following a previously reported protocol [29,30] with some modifications detailed elsewhere [19]. The final concentrations of antibiotics used for selecting *A. vinelandii* transformants on Burk’s sucrose plates were as follows: gentamicin (Gm) 1 µg ml^−1^; tetracycline (Tc) 30 µg ml^−1^; kanamycin (Km), 1 µg ml^−1^.

### Standard techniques

Genomic DNA isolation was performed using the Bacterial DNA preparation Kit, following the manufacturer’s instructions (Jena Bioscience). High-fidelity Phusion DNA polymerase (Thermo Fischer Scientific) was used for all PCR reactions, and when necessary, the products were confirmed by DNA sequencing. DNA sequencing was conducted with fluorescent dideoxy terminators using a cycle sequencing method and a 3,500xl analyzer (Applied Biosystems).

### Analytical methods

Protein concentration was determined using the Lowry method [31]. The activity of β-galactosidase in *A. vinelandii* cells was measured as previously reported [19]. Alginate concentration was determined as described [15], using the spectrophotometric determination of uronic acids with carbazole [32]. All experiments were conducted in triplicate, and the results presented are averages of the independent runs. Statistical analysis was performed using a Student’s *t*-test (*P* ≤ 0.05).

### Construction of mutants

The *fleQ* mutant was constructed by PCR amplification of a 2362 bp DNA fragment containing the *fleQ* gene, using chromosomal DNA from strain AEIV as template and the primer pairs FleQ-F (5′-GGC TTG GCT GGA AGC ATT G-3′) and FleQ-R (5′-GGC AGT CAG CAC ACG ATA G-3′). The PCR product was subsequently cloned into the vector pJET1.2/Blunt (Thermo Fisher), rendering plasmid pLA62. A SphI-EcoRV *fleQ* internal fragment of 1229 bp from plasmid pLA62 was replaced by a Gm cassette, released with SmaI endonuclease from plasmid pBSL190 [33]. Before cloning the resistance cassette, the SphI-EcoRV-excised pLA62 plasmid was made bluntly using the Klenow enzyme. The resultant plasmid was named pLA498 (Δ*fleQ*::Gm). The orientation of the resistance cassettes in plasmid pLA498 matches that of *fleQ*, preventing polar effects on downstream genes. Plasmid pLA498, linearized with ScaI endonuclease, was used to transform competent AEIV cells. The resulting Δ*fleQ* mutant was designated CLAM522 (Δ*fleQ*::Gm).

Genetic complementation of the Δ*fleQ*::Gm mutant was conducted by integrating a wt copy of *fleQ* into the genome of CLAM522. For this purpose, a 2362 bp DNA fragment containing the *fleQ* gene and its regulatory region was cloned into the pUMATc5′−3′ (Tc) vector, which had been previously excised with SmaI [34], resulting in plasmid pUMA-fleQ. This plasmid was then linearized with ScaI and introduced into CLAM522 via transformation. After double homologous recombination, the *fleQ* gene was integrated into the *A. vinelandii* chromosome within a neutral *locus* (*melA*). The complemented strain (Δ*fleQ*/*fleQ+*) was designated SM654.

The *algD-lacZ* (Km^r^) transcriptional fusion present in strain A2 [35] was used to assess the influence of intracellular levels of c-di-GMP or FleQ on *algD* transcription. Competent A2 cells were transformed with chromosomal DNA from the Δ*fleQ* (Gm^r^), DGC+ (Tc^r^) or PDE+ (Tc^r^) strains, followed by selection of transformants in the presence of the corresponding antibiotic. The resulting strains were designated JG621, JG623 and JG628, respectively.

### Real-time qPCR

The relative mRNA levels of the *algD* gene and the *algE1* to *algE6* (*algE1-6*) genes were determined by RT-qPCR, as previously described [16], using total RNA extracted from cells cultivated in Burk’s sucrose (vegetative conditions) or Burk’s *n*-butanol medium (encysting conditions) for 24 h. Total RNA was extracted as reported [36].

The primer pairs used were algD-RT3-F (5′-TTCGGACTGGGCTATGTAGG-3′)/algD-RT3-R (5′-GCCCTGATTGATCATGTCG-3′) for *algD* and FwRT-algE1-6 (5′-CACGAGCAGACCATCAACCTG-3′)/RvRT-algE1-6 (5′-ATGTTGAAGCCGTGGCGGTCGTTG-3′), for *algE1-6*.

Relative levels of *algD* and *algE1-6* were determined by comparing the amount of each mRNA using *gyrA* (Avin_15810) mRNA as an internal control. Three biological replicates (independent cultures) were performed, with three technical replicates for each. The quantification technique used to analyse the data was the 2^−ΔΔCT^ method [37].

### Identification of potential FleQ binding sites

Thirteen sequences identified as FleQ binding sites in *P. aeruginosa* [38] were used to build the position-specific scoring matrix and sequence *logo* of FleQ using MEME (*Multiple Em for Motif Elicitation*), with one occurrence per sequence being searched [39]. The position-specific scoring matrix was used as input to the FIMO (*Find Individual Motif Occurrences*) algorithm to search for FleQ binding sites in the 5′ UTR gene sequences of *A. vinelandii* DJ strain [40].

The 5′ UTR gene sequences of *A. vinelandii* were obtained using the bedtools getfasta command from the BedTools toolkit [41], encompassing 400 nucleotides upstream and 50 nucleotides downstream of the genomic start coordinate of each gene found in the *A. vinelandii* DJ annotation file.

### Expression and purification of the His-FleQ protein

FleQ was purified as a recombinant 6×His-tagged protein at the N-terminus (His-FleQ). The *fleQ* gene was PCR amplified using the primer pair fleQ-F-NdeI (5′-CATATGATGTGGCGTGACATAAAAATCCTCC-3′)/fleQ-R-BamHI (5′-GGATCCCGCAGCGAGGTTTAACAC-3′) and cloned into pET-28a vector, which had been previously digested with NdeI and BamHI endonucleases, leaving an N-terminal His_6_ tag. The resulting plasmid was named pET-FleQ.

The BL21 (DE3) *E. coli* strain (Invitrogen) carrying pET-FleQ was grown in LB Km at 37 °C until an OD_600_ of 0.6. At this point, 0.05 mM of IPTG (isopropyl-β-d-thiogalactopyranoside) was added to induce His-FleQ expression, and the culture was incubated at 15 °C for 16 h. Cells were harvested by centrifugation at 4,000×*g* for 10 min, washed once with cold lysis buffer (50 mM NaH_2_PO_4_, 300 mM NaCl, 10 mM imidazole [pH 8]; pH 8) and disrupted by sonication in the same buffer. The supernatant was recovered by centrifugation at 4,000×*g* for 30 min at 4 °C. The crude extract was loaded into a Ni-NTA agarose column previously equilibrated with lysis buffer. The column was washed ten times with washing buffer (50 mM NaH_2_PO_4_, 300 mM NaCl, 20 mM imidazole [pH 8]; pH 8), and the protein was eluted using elution buffer I (50 mM NaH_2_PO_4_, 300 mM NaCl, 250 mM imidazole [pH 8]; pH 8). The protein was then concentrated using an Amicon Ultra 50K device (Merck Millipore) and stored in elution buffer II (20 mM Tris-HCl [pH 8], 300 mM NaCl, 20% glycerol). Protein concentration was determined using the Bradford assay with bovine serum albumin (BSA) as standard. SDS-PAGE confirmed the purification of His-FleQ.

### Electrophoretic mobility shift assay

The electrophoretic mobility shift assays (EMSAs) were performed as previously described, using a non-radioactive method [42,43]. DNA fragments spanning the complete regulatory region of *algD* or only the distal or proximal region were PCR amplified using the primer pairs: complete fragment, algD-EMSA-F1.21 (5′-TACGGCAATCCCATTGCTG-3′)/palgD-R (5′-CCGAAAATGCTGATACGC-3′); distal region algD-EMSA-F1.21 (5′-TACGGCAATCCCATTGCTG-3′)/palgD-EMSAR2 (5′-TGGTCCTTCCATGGGTTAC-3′); proximal region, palgD-EMSAF3 (5′-CGAACATGGATCGCTTGAG-3′)/palgD-R (5′-CCGAAAATGCTGATACGC-3′). Chromosomal DNA of strain AEIV was used as DNA template. Binding reactions were performed by mixing 100 ng of the DNA fragment with increasing concentrations of purified His-FleQ protein in binding buffer (10 mM Tris [pH 8], 8 mM magnesium acetate tetrahydrate, 50 mM KCl, 10 µg ml^−1^ bovine serum albumin and 5% glycerol) in a total volume of 20 µl. Binding reactions were incubated at room temperature for 20 min before being loaded onto 6% non-denaturing acrylamide gels and electrophoresed at 60–80 V in 0.5×−1× Tris-Borate-EDTA (TBE) buffer. As a negative control, the regulatory region of the *Salmonella* Typhimurium *eutR* or *yebF* gene was included. These fragments of 558 bp or 136 bp, respectively, were PCR amplified using the primer pairs F2eutR-EcoRI (5′-CTTGAATTCGTTTGCTCAGTCATCAAGTGC-3′)/R2eutR-BamHI (5′-CTTGGATCCCGCTGATGAACATTGTCCACC-3′) or yebF-FwEcoRI (5′-GCGAATTCGGACGCCGCGAGTAAAACG-3′)/yebF-RvBamHI (5′-CCTGGATCCAGCAGGCTCAACAACGCTCC-3′). Migration of the DNA bands was visualized by staining with ethidium bromide (EtBr) under UV light.

### Detection of AlgE C-5 epimerases

The Western blot was performed using anti-AlgE4 antibodies to detect AlgE mannuronan C-5 epimerases on the surface of differentiated cells, following a previously described protocol [44]. Protein extracts from the surface of differentiated cells were prepared as described in [16]. For comparison purposes, the number of cells used to prepare the extracts of cell surface-associated proteins was normalized beforehand. Therefore, the detected bands can be reliably compared among the different strains.

## Results

### *algD* transcription is modulated by the levels of c-di-GMP

**Fig. 1.**
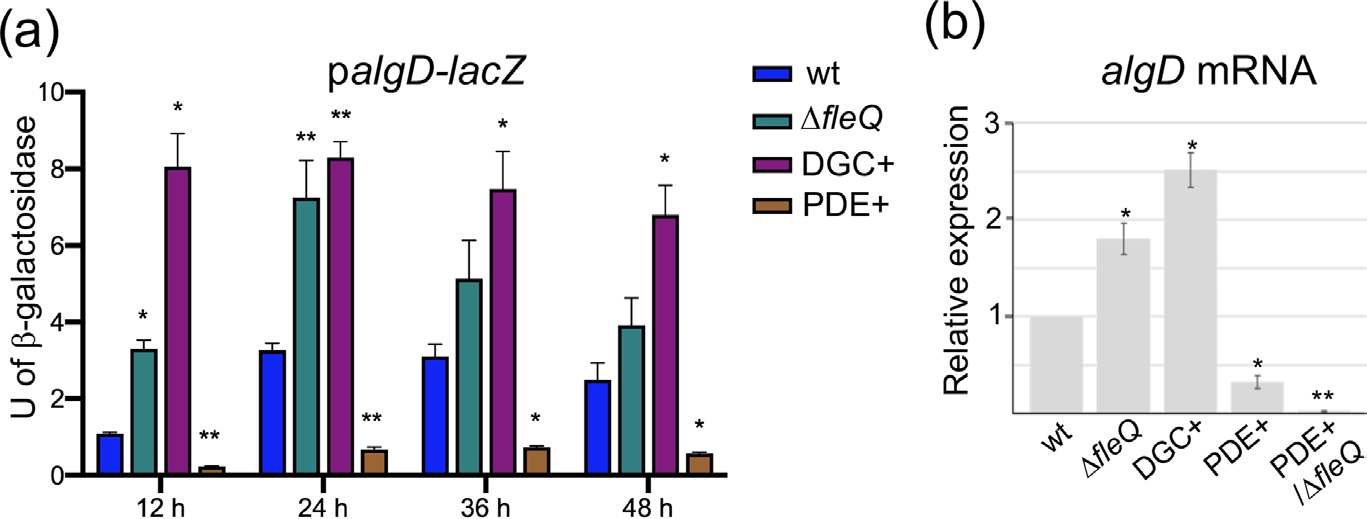
*algD* transcription responds to the levels of c-di-GMP and is under the control of FleQ. (**a**) Kinetics of *algD* transcription using an *algD-lacZ* transcriptional fusion in strains wt and Δ*fleQ*, or in strains showing high (DGC+) or reduced (PDE+) levels of c-di-GMP. The strains were grown in liquid Burk’s-sucrose medium for the indicated time. (**b**) *algD* mRNA quantification by RT-qPCR using RNA extracted from strains wt, Δ*fleQ*, DGC+, PDE+ and PDE+/Δ*fleQ* cultivated in Burk’s sucrose liquid medium for 24 h. The bars for standard deviation from three independent experiments are shown. Significant differences were analysed by *t*-test. Statistical significance is shown. *P < 0.01; **P < 0.001.

To investigate whether the expression of *algD* is controlled by c-di-GMP, its transcription was monitored using an *algD-lacZ* fusion in genetic backgrounds with artificially altered levels of c-di-GMP. This was achieved by overexpressing the *A. vinelandii* DGC *Av*GReg (DGC+) or the PDE Avin_50640 (PDE+), which, as shown in a previous study, results in a fivefold increase or at least a 100-fold reduction in c-di-GMP levels, respectively, relative to the wt strain [15]. Strikingly, in the DGC+ genetic background, *algD* transcription was markedly high along the growth curve, and this effect was more pronounced during the logarithmic phase (at 12 h) (Fig. 1a). In contrast, *algD* transcription was almost abrogated in strain PDE+. This data was further supported by RT-qPCR analysis, using total RNA extracted from cells cultivated in Burk’s sucrose medium for 24 h. Transcripts of *algD* were 2.5-fold higher and threefold lower in the DGC+ and PDE+ strains, respectively, relative to the wt (Fig. 1b). These results revealed a positive effect of c-di-GMP on *algD* transcription, raising the question about the molecular mechanism underlying this regulation.

### FleQ binding sites prediction in the *A. vinelandii* genome

The *A. vinelandii* genome encodes a FleQ protein of 501 amino acids showing 71% identity when compared to that of *P. aeruginosa*. Motifs important for the binding of dimeric c-di-GMP, interaction with RpoN or the active sites for the AAA+ domain are conserved between the two proteins (Fig. S1, available in the online version of this article). Similarly, sequences of the FleQ HTH domains and structure prediction by AlphaFold are conserved between *A. vinelandii* and *P. aeruginosa* (Fig. S2). It is of interest to note that a 21 amino acid motif is present at the C-terminus of FleQ proteins from the *Azotobacter* but not from the *Pseudomonas* genus (Fig. S2). However, the functional relevance of this feature is not known.

**Fig. 2.**
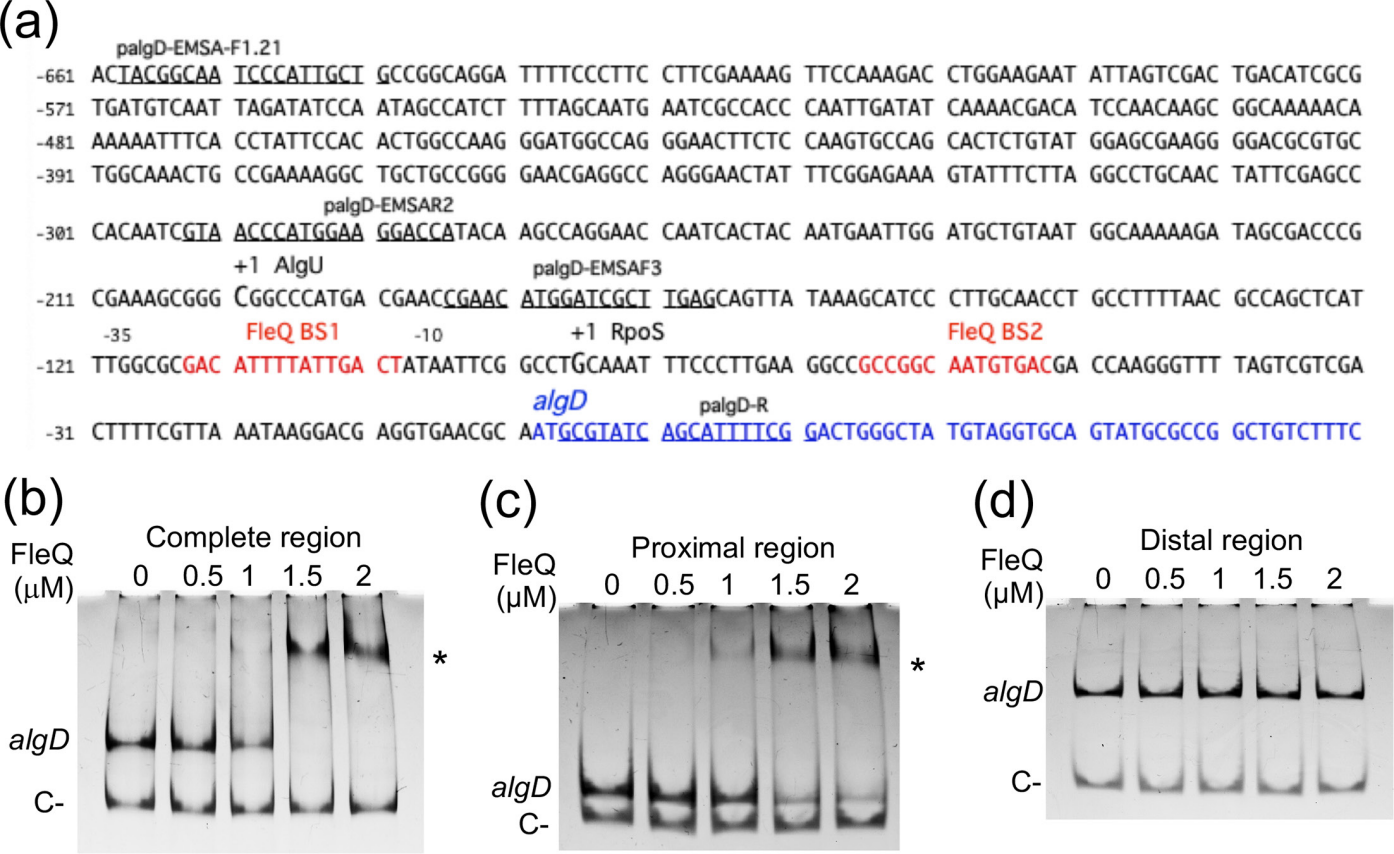
FleQ binds to the *algD* regulatory region. (**a**) DNA sequence of the *algD* regulatory region. The transcription start sites (+1) previously identified for the RpoS and AlgU promoters are indicated, along with the two predicted FleQ binding sites (BS1 and BS2), which overlap or are close to the RpoS promoter. The structural region of *algD* is shown in blue. The oligonucleotides used to amplify the DNA fragments for the EMSAs are underlined. The numbers on the left indicate the nucleotide position relative to the translational start codon. Non-radioactive EMSAs were performed to detect FleQ binding to the complete regulatory region of *algD* (nucleotides −659 to +20) (**b**); or to the proximal (nucleotides −186 to +20) (**c**), or distal (nucleotides −659 to −276) (**d**) region. The DNA fragments were incubated with increasing concentrations of His-FleQ. The asterisk in (**b**) and (**c**) denotes the formation of an *algD*-FleQ complex. As negative controls (C−), fragments of the *Salmonella* Typhimurium *eutR* (558 bp) (**b**) or *yebF* (136 bp) (**c, d**) genes were included. The migration of the DNA fragments was visualized by staining with ethidium bromide.

A MEME/FIMO analysis was conducted as described in Methods, aimed at identifying potential targets of FleQ in the *A. vinelandii* DJ genome (Table S1). Given the conservation of the HTH domain between the FleQ proteins from *A. vinelandii* and *P. aeruginosa*, this analysis was carried out using *P. aeruginosa* experimentally demonstrated FleQ binding sites [38]. A total of 226 putative FleQ binding sites were identified with a *P*-value < 0.0001 (Table S1), among them, a binding site within the regulatory region of *algD* (*P*-value = 4.30E−05), overlapping its RpoS-dependent promoter (BS1) (Fig. 2a). A second potential FleQ binding site was also identified (BS2), 32 bp apart with a *P*-value slightly lower (*P*-value = 4.73E−4). Thus, the role of FleQ on alginate production and *algD* transcription was investigated.

### FleQ inhibits alginate production

**Fig. 3.**
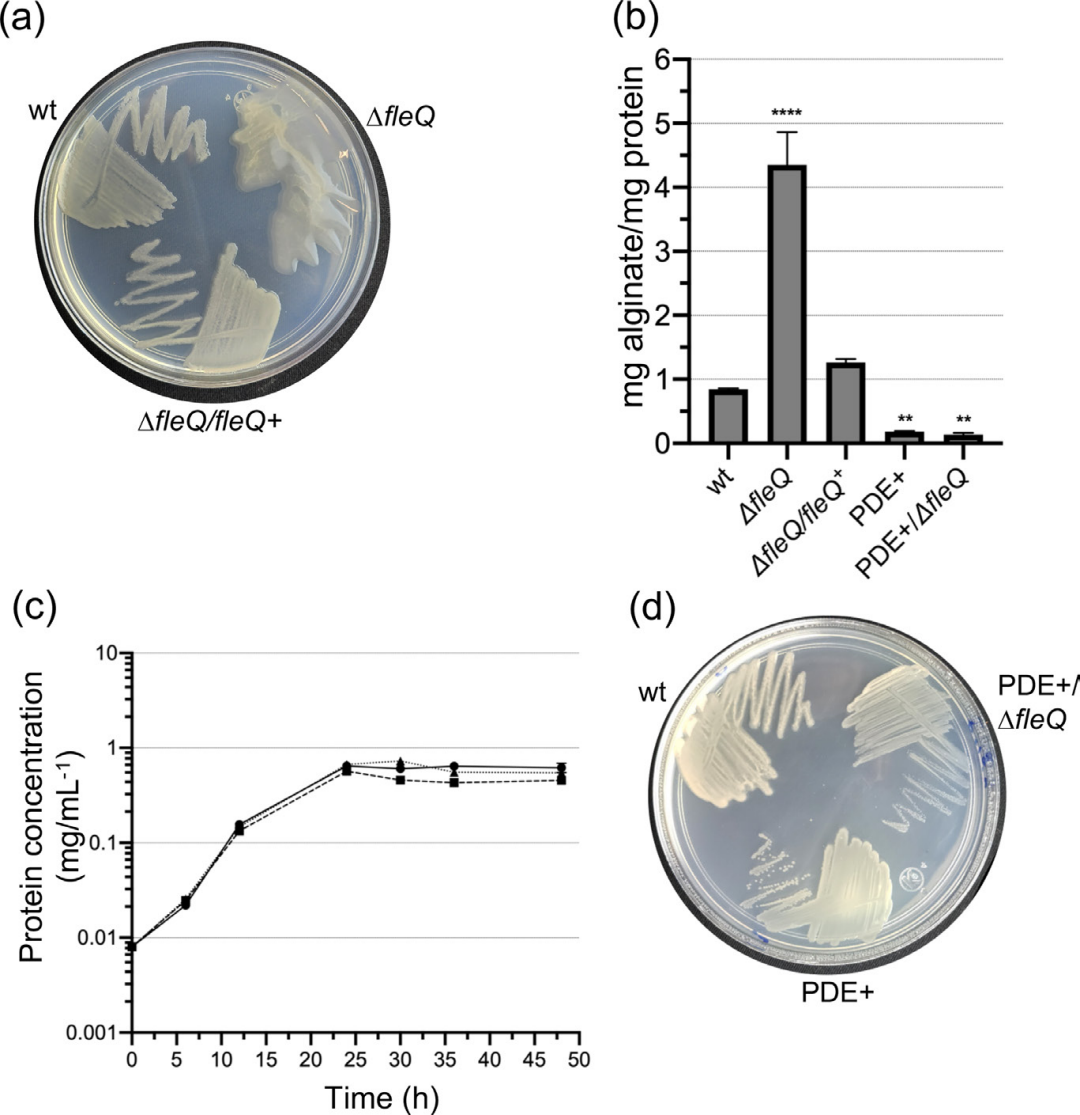
FleQ represses alginate production. (**a**) Growth of the wild-type strain (wt), the Δ*fleQ* mutant and its derivative Δ*fleQ*/*fleQ*+ on Burk’s sucrose plates, incubated for 48 h. The hyper mucoid colony phenotype of strain Δ*fleQ* is readily apparent. (**b**) Quantification of the alginate produced by *A. vinelandii* strains grown in Burk’s sucrose liquid medium for 48 h. The bars for standard deviation from three independent experiments are shown. Significant differences were analysed by *t*-test. Statistical significance is shown. ***P < 0.0001. (**c**) Growth kinetics of wt strain (solid line), the Δ*fleQ* mutant (dashed line) and of its complemented strain Δ*fleQ/fleQ+* (dotted line) in Burk’s sucrose medium. (**d**) Growth of strains wt, PDE+ and PDE+/Δ*fleQ* on Burk’s sucrose plates, incubated for 48 h.

To explore the role of FleQ, a Δ*fleQ* deletion mutant was constructed in *A. vinelandi*. A hyper mucoid colony phenotype was readily apparent in the Δ*fleQ* mutant, suggesting a negative effect of FleQ on alginate production (Fig. 3a). Indeed, alginate production by the Δ*fleQ* mutant increased fourfold, relative to the wt strain, when cultured in Burk’s sucrose liquid media (Fig. 3b). A complemented Δ*fleQ* derivative strain (Δ*fleQ* /*fleQ*^+^), carrying a wt copy of *fleQ* under its own promoter showed wt levels of alginate, thus confirming the negative role of FleQ in this biosynthetic pathway (Fig. 3a, b). The growth of the Δ*fleQ* mutant was slightly decreased with respect to the wt and the Δ*fleQ*/*fleQ^+^* complemented strain, likely due to its alginate over-producing phenotype (Fig. 3c).

### FleQ inhibits *algD* transcription

Given the negative effect of FleQ on alginate production, its influence on *algD* transcription was investigated. The *algD-lacZ* transcriptional fusion was used to evaluate the effect of FleQ on *algD*, at different times of the *A. vinelandii* growth curve. As shown in Fig. 1a, consistently higher levels of β-galactosidase were detected in the absence of FleQ, mostly during the logarithmic phase of growth (12 and 24 h). This result was further supported by RT-qPCR using mRNA extracted from strains wt and Δ*fleQ* cultured in liquid Burk’s sucrose medium for 24 h. The relative mRNA levels were 1.8-fold higher in the Δ*fleQ* mutant (Fig. 1b), confirming the negative effect of FleQ on *algD* transcription.

### FleQ is a direct repressor of *algD* transcription

EMSAs were conducted to determine whether FleQ directly binds to the regulatory region of *algD*, as predicted by the MEME/FIMO analysis. To this end, we used the purified His-FleQ protein and various DNA fragments spanning the *algD* regulatory region (Fig. 2a). A fragment corresponding to the entire *algD* regulatory region (679 bp) exhibited reduced mobility in the presence of His-FleQ, indicating the formation of a DNA–protein complex (Fig. 2b). Similarly, a 206 bp fragment containing the RpoS promoter and the putative FleQ binding sites was shifted in the presence of increasing concentrations of His-FleQ (Fig. 2c), suggesting that these FleQ binding sites are functional. The binding of FleQ appears to be specific, as FleQ did not affect the migration of DNA fragments used as negative controls (Fig. 2b–d). In contrast, a 383 bp fragment corresponding to the distal part of the *algD* regulatory region was not shifted in the presence of His-FleQ (Fig. 2d). As expected, *A. vinelandii* His-FleQ also retarded a DNA fragment carrying the * P. aeruginosa pelA* gene, which was included as a positive control in these assays (Fig. S3). These results confirm that FleQ can recognize the regulatory region of *algD*.

### The absence of FleQ is not sufficient to relieve repression of *algD* at low c-di-GMP

Our data show that c-di-GMP induces expression of *algD* and that FleQ directly represses *algD* transcription, suggesting that c-di-GMP counteracts the repression exerted by FleQ. We investigated whether *algD* expression could be restored under low c-di-GMP levels in the absence of FleQ. To this end, a Δ*fleQ* mutant was constructed in a genetic background that overexpresses the PDE Avin_50640 (PDE+), leading to a marked reduction in c-di-GMP levels. However, in the PDE+/Δ*fleQ* strain, *algD* expression, quantified by RT-qPCR at 24 h of growth, was not restored to wt levels (Fig. 1b). This result is consistent with the non-mucoid colony phenotype of this strain on solid media (Fig. 3d), which is similar to that of the PDE+ strain. In agreement with their colony phenotype, both the PDE+ and PDE+/Δ*fleQ* strains exhibited only basal levels of uronic acid detection (Fig. 3b), as previously described for the alginate minus mutant *algD,* of *A. vinelandii* [15]. These findings suggest the existence of an additional level of *algD* regulation by c-di-GMP that is independent of FleQ.

### Expression of *algE1-6* genes responds to the c-di-GMP levels

**Fig. 4.**
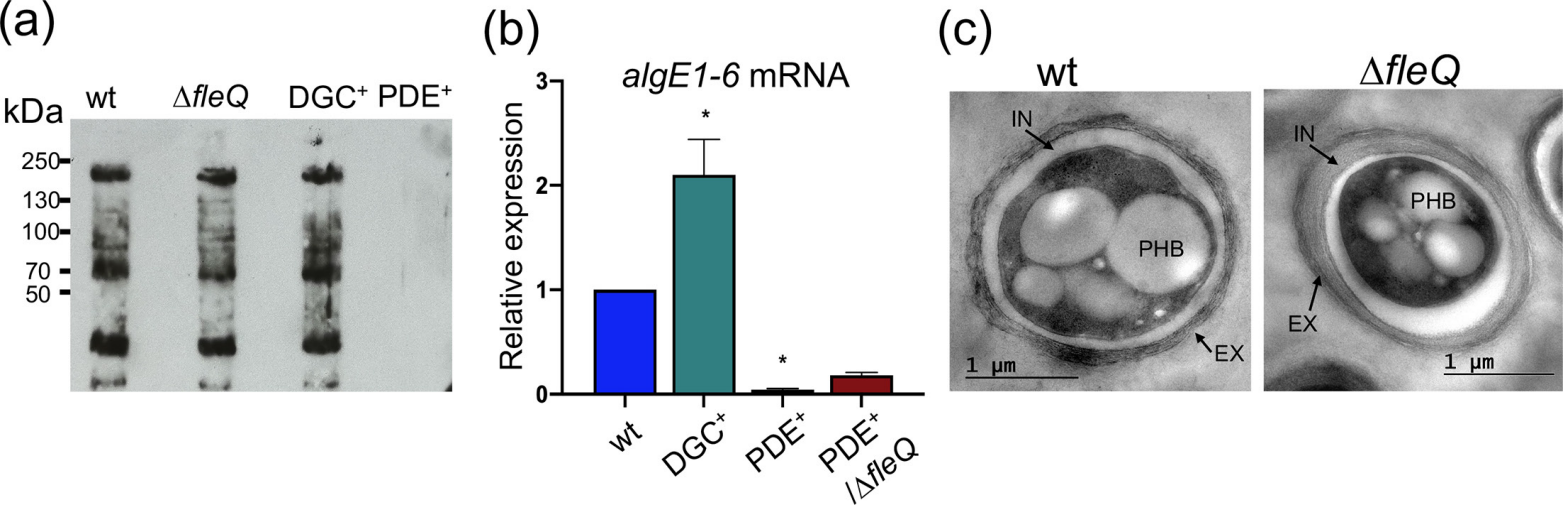
The absence of FleQ does not impair *algE1-6* expression or mature cyst formation. (**a**) Western blot to detect AlgE mannuronan epimerases over the surface of wt, Δ*fleQ*, DGC+ or PDE+ cells, after 5 days on Burk’s *n*-butanol plates. Anti-AlgE4 antibody was used as the primary antibody. (**b**) Determination of the relative abundance of *algE1-6* mRNA by RT-qPCR in cells grown for 48 h in Burk’s sucrose medium. The bars for standard deviation from three independent experiments are shown. Significant differences were analysed by *t*-test. Statistical significance is shown. *P < 0.01. (**c**) Electron micrographs of *A. vinelandii* cysts formed by the wt strain and the Δ*fleQ* mutant, after 5 days of differentiation on Burk’s *n*-butanol plates. The cyst capsule is clearly observed, which is composed of two laminated alginate layers, the exine (EX) and the intine (IN). Poly-B-hydroxybutyrate (PHB) granules are also observed.

In *A. vinelandii*, the DGC MucR was found essential for the transcription of the *algE1-6* genes, encoding alginate C-5 epimerases [16]. However, a direct link between c-di-GMP and *algE1-6* expression had not been established. To address this question, detection of the AlgE1-6 proteins on the surface of differentiated cells, in the genetic backgrounds of DGC+ or PDE+ strains, was conducted by Western Blot. The higher levels of c-di-GMP in strain DGC+ did not change the detection pattern of the epimerases attached to the cyst surface (Fig. 4a); in contrast, reduced levels of c-di-GMP abolished the detection of these enzymes on the surface of PDE+ cells. This effect seems to be at the transcriptional level, as *algE1-6* transcripts were almost undetectable in strain PDE+ (Fig. 4b) but were twofold higher in strain DGC+, confirming the positive role of c-di-GMP on the expression of *algE1-6* genes. Transcripts of *algE1-6* genes were simultaneously detected as described in Methods, using a single pair of primers previously reported [45], and able to anneal to *algE1-6* genes due to the modularity of the encoded proteins [46].

### FleQ is not essential for mature cyst formation

Next, we investigated if FleQ could be an intermediary in the regulation of *algE1-6* by c-di-GMP. Western Blot assays revealed that when cultivated under encystment-induced conditions, the Δ*fleQ* mutant showed a pattern of AlgE1-6 detection (Fig. 4a) and an accumulation of *algE1-6* transcripts like that of the wt strain (1.11 ± 0.09 for Δ*fleQ* vs 1.0 for wt). These results agreed with a frequency (%) of mature cyst formation by Δ*fleQ* at levels comparable to those shown by the wt strain (12.7% ± 3.2 vs 10.5% ± 2.0, respectively). The Δ*fleQ* cysts showed a typical morphology, with the exine and intine layers of the alginate protecting coat clearly visible (Fig. 4c). These data ruled out a positive essential role of FleQ on the expression of *algE1-6* genes and during mature cyst formation.

We also investigated a potential repressing role of FleQ on *algE1-6* expression. Low levels of c-di-GMP in strain PDE+ drastically reduced expression of *algE1-6* genes and detection by Western Blot of the corresponding proteins on the surface of the differentiated cells (Fig. 4a, b). To reveal a potential repressing effect of FleQ on *algE1-6* expression under reduced c-di-GMP levels, detection of *algE1-6* mRNA was conducted in the double mutant PDE+/Δ*fleQ*. As shown in Fig. 4b, expression of *algE1-6* genes was not restored in this genetic background, confirming that FleQ is not an intermediary in the control of *algE1-6* transcription by the second messenger c-di-GMP.

## Discussion

In contrast to the role of alginate in *P. aeruginosa*, in *A. vinelandii*, alginate is part of the envelope of differentiated cells resistant to desiccation [2]. This fact could be the reason for the differing transcriptional regulation of *algD* in these two bacteria. While in *P. aeruginosa*, transcription of *algD* is driven solely by an AlgU-dependent promoter, in *A. vinelandii,* it is transcribed from at least two well-characterized promoters, dependent on AlgU and RpoS [4,47].

In this study, we demonstrated that *algD* transcription in *A. vinelandii* positively responds to c-di-GMP levels and that FleQ represses *algD* transcription by directly binding to its regulatory region. This finding aligns with the predicted FleQ binding site overlapping the *algD* RpoS promoter. In *P. aeruginosa*, FleQ represses *pel* operon transcription at low c-di-GMP levels, whereas at high levels, upon binding to c-di-GMP, FleQ activates *pel* transcription [24]. At low c-di-GMP levels, FleQ binds to two sites located 35 bp apart and flanking the −10 and −35 region of the *pelA* promoter. Upon binding to these sites, FleQ together with FleN, induces DNA backbone distortion, preventing RNAP binding to the *pelA* promoter [22,24].

In *A. vinelandii,* a second FleQ binding site was also identified, 32 bp apart from the first one. Therefore, it is tempting to propose a repressing model for FleQ-mediated control of *A. vinelandii algD*, similar to the regulation of *P. aeruginosa pelA* under low c-di-GMP levels. Supporting this idea, an *A. vinelandii* Δ*fleN* mutant exhibits an alginate-overproducing phenotype, similar to that of the Δ*fleQ* mutant, which is reflected in the mucoid colony phenotype compared to the wt strain (Fig. S4). However, the precise molecular mechanism underlying the repression of *algD* by FleQ, and possibly FleN, in *A. vinelandii* remains to be investigated.

Notably, reduced c-di-GMP levels almost completely abolished *algD* expression throughout the growth curve (Fig. 1a), suggesting that not only the RpoS-dependent promoter, but also the AlgU-dependent promoter is regulated by intracellular c-di-GMP levels, likely through a multilayer effect. Indeed, the absence of FleQ under reduced levels of c-di-GMP did not restore *algD* expression or alginate production, revealing an additional level of regulation by this second messenger. In *P. aeruginosa*, c-di-GMP has been shown to exert a positive regulatory effect on the AlgU-dependent *algD* promoter. However, the molecular mechanism of this effect remains unknown, as the transcriptional factors reported to directly activate *algD* expression (such as AlgR, AlgB and AmrZ) do not bind c-di-GMP [48].

In this study, we confirmed that transcription of the *algE1-6* genes, encoding alginate C-5 epimerases necessary for cyst capsule assembly, is positively regulated by c-di-GMP. However, our data suggest that FleQ is not involved in this regulation, as *algE1-6* expression was unaffected in the Δ*fleQ* mutant, and expression at low c-di-GMP in the absence of FleQ was not restored to wt levels (Fig. 4a, b). Although FleQ was not essential for the formation of mature, desiccation-resistant cysts, we cannot rule out a regulatory role for this transcription factor in *A. vinelandii* encystment. Ongoing research aims to identify additional processes regulated by FleQ and c-di-GMP during vegetative growth or encystment in *A. vinelandii*, with the goal of optimizing the production of tailor-made alginates or increasing cyst formation frequency to enhance inoculant formulations for sustainable agriculture.

## Supplementary material

10.1099/mic.0.001556 Uncited Supplementary Material 1.

## Abbreviations

c-di-GMP: bis(3′,5')-cyclic dimeric guanosine monophosphate
DGC: diguanylate cyclases
EMSA: electrophoretic mobility shift assay
FIMO: Find Individual Motif Occurrences
MEME: Multiple Em for Motif Elicitation
PDE: phosphodiesterases
wt: wild-type
