# Supplementary material for: The c-di-GMP effector FleQ controls alginate production by repressing transcription of algD in Azotobacter vinelandii
Source: Microbiology (Reading). 2025 Apr 24;171(4):001556. doi: 10.1099/mic.0.001556 (PMC12022260; doi:10.1099/mic.0.001556)

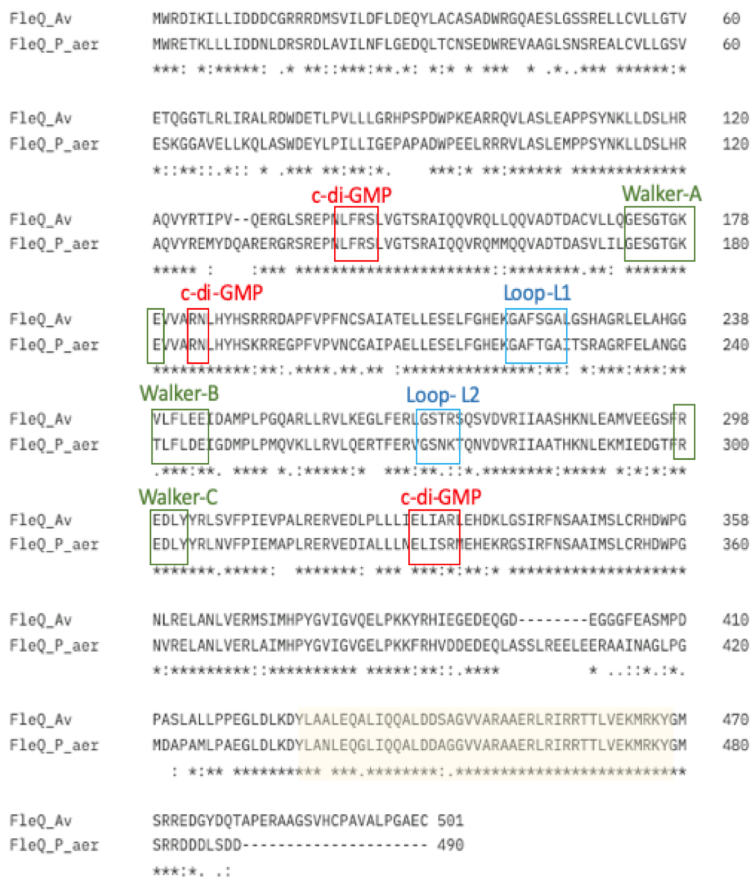

Fig. S1. Sequence alignment of FleQ. FleQ alignment from *A. vinelandii* AEIV (Av) and *P. aeruginosa* PAO1 (P_aer). Motifs for c-di-GMP binding, interaction with sigma 54, or active sites for the AAA+ domain are indicated in red, blue or green boxes. The HTH domain is highlighted in yellow.

A

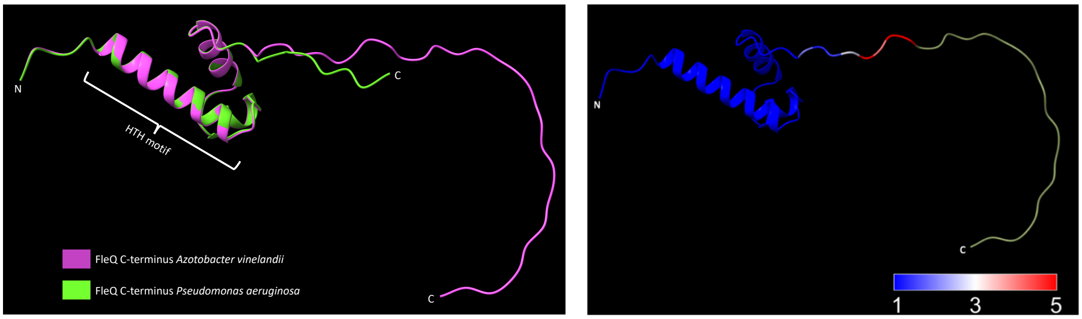

B

```
FLEQ_AV_DJ       PASLALLPPEGLDLKDYLAALEQALIQQALDDSAGVVARAAERLRIRRTTLVEKMRKYGM  470
FLEQ_AV_AEIV     PASLALLPPEGLDLKDYLAALEQALIQQALDDSAGVVARAAERLRIRRTTLVEKMRKYGM  470
FLEQ_A_BEIJ      PSSLALLPPEGLDLKDYLAALEQTLIQQALDDSAGVVARAAERLRIRRTTLVEKMRKYGM  470
FLEQ_A_CRO       PATLALLPPEGLDLKDYLAALEQTLIQQALDDSAGVVARAAERLRIRRTTLVEKMRKYGM  470
FLEQ_P_FL        FSASALLPPEGLDLKDYLGGLEQGLIQQALDDANGIVARAAERLRIRRTTLVEKMRKYGM  480
FLEQ_P_PU        FSNHAMLPPEGLDLKDYLGSLEQGLIQQALDDANGIVARAAERLRIRRTTLVEKMRKYGM  480
FLEQ_P_AER       MDAPAMLPAEGLDLKDYLANLEQGLIQQALDDAGGVVARAAERLRIRRTTLVEKMRKYGM  480
FLEQ_P_RES       IASPALLPPEGLDLKDYLGNLEQGLIQQALDDASGVVARAAERLRIRRTTLVEKMRKYGM  480
FLEQ_P_OT        VSAPAMLPPEGLDLKDYLGNLEQELIQQALDDAAGVVARAAERLRIRRTTLVEKMRKYGM  480
FLEQ_P_IND       MTSPAMLPPEGLDLKDYLGNLEQGLIQQALDDAGGIVARAAERLRIRRTTLVEKMRKYGM  480
                     *:** *********. *** ********: *:************************

FLEQ_AV_DJ       SRREDGYDQTAPERAAGSVHCPAVALPGAEC       501
FLEQ_AV_AEIV     SRREDGYDQTAPERAAGSVHCPAVALPGAEC       501
FLEQ_A_BEIJ      SRREDGGNAVGPGAFVAVDNRSAVAPPGAEC       501
FLEQ_A_CRO       GRREDGDDQPDPESAAAAGHRSAVAPPGAEC       501
FLEQ_P_FL        SRAGGDEQAD---------------------       490
FLEQ_P_PU        SRQGGEDQAED--------------------       491
FLEQ_P_AER       SRRDDDLSDD---------------------       490
FLEQ_P_RES       SRRDEELAEE---------------------       490
FLEQ_P_OT        SRRDEDMAED---------------------       490
FLEQ_P_IND       SRRDDEDQPED--------------------       491
                 .*
```

Fig. S2. Alignment of the C-terminal region of FleQ. A. Structure prediction using the AlphaFold program of the C-terminal region of FleQ from *A. vinelandii* and *P. aeruginosa*, containing the HTH motif. Left panel, superposition of the C-terminus. Right panel, a RMSD value of 0.431 angstroms was obtained considering only the 57 pruned atoms pairs from the HTH domain. **B.** Sequence alignment of the C-terminal region of FleQ from *A. vinelandii* DJ (AV_DJ), AEIV (AV_AEIV), *Azotobacter Beijerinckia* (A_BEIJ), *Azotobacter croococcum* (A_CRO), *Pseudomonas fluorescens* (P_FL), *Pseudomonas putida* (P_PU), *P. aeruginosa* (P_AER), *Pseudomonas resinovorans* (P_RES), *Pseudomonas otitidis* (P_OT), *Pseudomonas indica* (P_IND). Protein alignment was conducted in Clustal Omega (MSA).

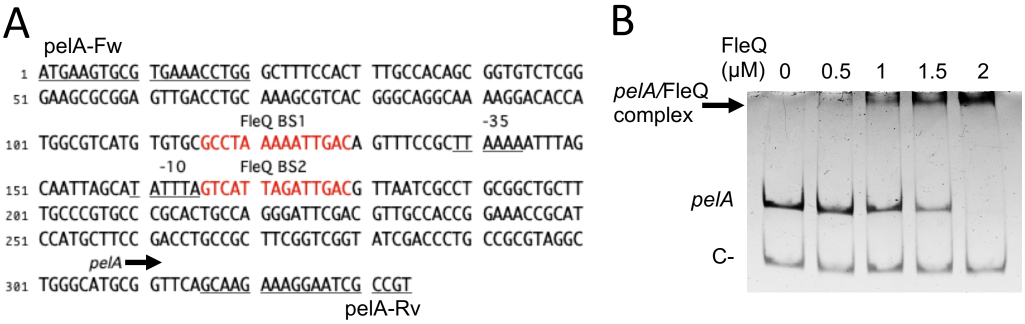

Fig. S3. ***A. vinelandii*** **FleQ binds to the** ***P. aeruginosa pelA*** **promoter. A.** DNA sequence of the *pelA* regulatory region. The -10 and -35 regions of the *pelA* promoter, as well as the FleQ binding sites previously reported by Baraquet et al. (2012), are shown. **B.** EMSAs to evaluate the binding of *A. vinelandii* His-FleQ to the *pelA* regulatory region. A 334 bp *pelA* fragment was PCR amplified using the primer pair pelA-fw/pelA-Rv depicted in panel A. 100 ng of DNA was incubated with increasing concentrations of His-FleQ. A 207 bp fragment of the *eutR* gene from S. Typhimurium was used as negative control (C-) and it was PCR amplified using the primer pair eutR-FwEcoRI (5’-CTTGAATTCGAACAACTCTTTTCTGACGGAG-3’)/ R2eutR-BamHI (5’-CTTGGATCCCGCTGATGAACATTGTCCACC-3’). The migration was visualized by staining with ethidium bromide.

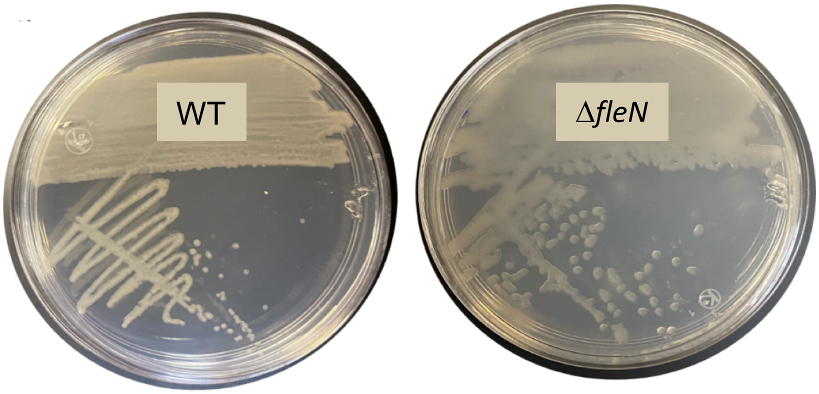

Fig. S4. The Δ*fleN* mutant shows an alginate-overproducing phenotype. Growth of the wild type strain (wt) and the Δ*fleN* mutant on Burk's-sucrose plates for 48 h. The Δ*fleN* mutant shows an hyper mucoid colony phenotype similar to that of mutant Δ*fleQ*.

S1 Table. List of potential FleQ binding sites in the genome of *A. vinelandii* detected by a MEME/FIMO analysis using experimentally identified *P. aeruginosa* FleQ binding sites.

*locus*_tag	start*	end	Distance to ATG codon	Strand	Score	p-val	q-val	Sequence	Gene	Product
Avin_05110	490958	490971	100	-	19.4388	5.72E-08	0.135	GTCAAAAAACCGAC		hypothetical protein
Avin_05120	490958	490971	349	-	19.4388	5.72E-08	0.123	GTCAAAAAACCGAC	*ompR*	two-component response regulator OmpR
Avin_37690	3814816	3814829	-20	+	17.2653	8.69E-07	0.761	GTCAAAAGATTGGC		conserved hypothetical protein
Avin_50730	5141247	5141260	188	+	17.1429	9.70E-07	0.761	GTCGGAAAATCGAC	*modA3*	molybdate ABC transporter, periplasmic molybdate-binding protein
Avin_52190	5334153	5334166	26	-	16.1633	2.27E-06	1	GTCGAAAAACTGGC	*atpH*	F1 sector of membrane-bound ATP synthase, delta subunit
Avin_12070	1160532	1160545	-22	+	15.5612	3.55E-06	1	GCCAGAAGATCGAC	*pilD*	type IV Pilus Prepilin peptidase, PilD
Avin_27160	2787068	2787081	-8	-	14.7857	6.09E-06	1	GTCATTTGATCGAC		Phytanoyl-CoA dioxygenase
Avin_47000	4770677	4770690	224	-	14.7245	6.37E-06	1	GTCGAAAAACTGTT	*rpoD*	RNA polymerase sigma factor, sigma70; RpoD
Avin_08290	787011	787024	27	+	14.6429	6.70E-06	1	GTCGGAAAACTGGC		conserved hypothetical protein
Avin_08300	787011	787024	227	+	14.6429	6.70E-06	1	GTCGGAAAACTGGC	*lrgA*	integral membrane protein, LrgA family
Avin_00420	41396	41409	355	-	14.551	7.17E-06	1	GTCGTTATATTGAC		GGDEF domain protein
Avin_17870	1774138	1774151	160	-	14.2755	8.44E-06	1	GTTATTAGATTGAC		conserved hypothetical protein
Avin_39680	4018322	4018335	190	-	14.2653	8.47E-06	1	GCCGGAAGATCGAC		Cytidine/deoxycytidylate deaminase-like protein
Avin_27790	2862291	2862304	126	+	14.2551	8.53E-06	1	GCCGAAAATCCGAC		UDP-glucose 6-dehydrogenase
Avin_27810	2862291	2862304	255	+	14.2551	8.53E-06	1	GCCGAAAATCCGAC		hypothetical protein
Avin_51880	5288801	5288814	94	+	14.1531	9.08E-06	1	GCCATAAAATTGAT		Superfamily I DNA and RNA helicases and helicase subunits-related protein
Avin_04180	399199	399212	-30	+	14.0408	9.68E-06	1	GCCAGAAGAGCGAC		hypothetical protein
Avin_04190	399199	399212	141	+	14.0408	9.68E-06	1	GCCAGAAGAGCGAC		hypothetical protein
Avin_51250	5210898	5210911	186	-	13.949	1.02E-05	1	GTCGGAAAACTCAC	*algE7*	Secreted bifunctional mannuronan C-5 epimerase/alginate lyase
Avin_01710	161252	161265	171	-	13.898	1.06E-05	1	GTCGCAAATGCGAC	*nifF*	Flavodoxin, nifF
Avin_29750	3076906	3076919	-17	-	13.8367	1.10E-05	1	GCCAGAAGTTCGAC	*lpdA*	dihydrolipoamide dehydrogenase
Avin_34840	3556028	3556041	105	+	13.7653	1.14E-05	1	GTCGGAAAACCGGC		ABC transporter, ATP-binding protein
Avin_11960	1147671	1147684	127	-	13.6429	1.22E-05	1	GCCGAAAAATCGGC	*clpB*	ATP-dependent protease
Avin_18580	1840518	1840531	240	-	13.5918	1.25E-05	1	GTCGGAAAATCCAC		conserved hypothetical protein
Avin_15090	1487526	1487539	383	+	13.5714	1.27E-05	1	GTCGCAAGATCGGC	*mhpR*	Bacterial regulatory protein, IclR family
Avin_18850	1872615	1872628	173	-	13.4184	1.38E-05	1	GCCAGAAAATCGGC		conserved hypothetical protein
Avin_18860	1872615	1872628	-40	-	13.4184	1.38E-05	1	GCCAGAAAATCGGC		NAD-dependent glutamate dehydrogenase
Avin_39180	3970876	3970889	205	+	13.3878	1.40E-05	1	GTCGAAAATTCCAC	*cspA*	Cold-shock-like protein
Avin_39190	3970876	3970889	-22	+	13.3878	1.40E-05	1	GTCGAAAATTCCAC		hypothetical protein
Avin_60180	1995846	1995859	112	-	13.051	1.68E-05	1	GTCGCAAGACCGGC	*tRNA*	tRNA-Asn
Avin_11330	1080697	1080710	-27	-	13.0408	1.69E-05	1	GTCGATTGTCCGAC		hypothetical protein
Avin_11340	1080697	1080710	51	-	13.0408	1.69E-05	1	GTCGATTGTCCGAC		Bacterial outer membrane porin
Avin_27550	2833221	2833234	-40	+	12.898	1.82E-05	1	GCCAGAAAACCGGC		Peptidase, S49 family
Avin_27570	2833221	2833234	216	+	12.898	1.82E-05	1	GCCAGAAAACCGGC		fructose-2,6-bisphosphatase
Avin_16040	1585672	1585685	357	-	12.8265	1.89E-05	1	GTCACTATTTTGTC		hypothetical protein
Avin_16050	1585672	1585685	157	-	12.8265	1.89E-05	1	GTCACTATTTTGTC		conserved hypothetical protein
Avin_16060	1585672	1585685	360	-	12.8265	1.89E-05	1	GTCACTATTTTGTC		hypothetical protein
Avin_19220	1917177	1917190	129	+	12.6735	2.03E-05	1	GTCGAAAGTTCCAC	*rnfE*	Electron transport complex, subunit E
Avin_35450	3618054	3618067	330	-	12.6224	2.09E-05	1	GTCGATAAAGCGTT		hypothetical protein
Avin_23460	2348107	2348120	81	-	12.5408	2.17E-05	1	GCCGAAAGACCGAT	*nasS*	nitrate/nitrite transport system substrate-binding protein ; NasS
Avin_35250	3602312	3602325	199	-	12.4898	2.23E-05	1	GTCGGAAGACTGTT		hypothetical protein
Avin_35260	3602312	3602325	15	-	12.4898	2.23E-05	1	GTCGGAAGACTGTT		hypothetical protein
Avin_35270	3602312	3602325	238	-	12.4898	2.23E-05	1	GTCGGAAGACTGTT		conserved hypothetical protein
Avin_44590	4509487	4509500	286	+	12.4796	2.24E-05	1	GCCGGAAAACTGGC		conserved hypothetical protein
Avin_39490	4000343	4000356	231	-	12.4286	2.29E-05	1	GTCAGATGATCGGC	*hom*	homoserine dehydrogenase
Avin_16060	1585949	1585962	83	-	12.4082	2.32E-05	1	GCCGAAAGACCGGC		hypothetical protein
Avin_30500	3160798	3160811	50	-	12.398	2.33E-05	1	GTCAGTAGTCCGGC	*ssuD*	alkanesulfonate monooxygenase
Avin_51880	5288681	5288694	-26	+	12.3367	2.41E-05	1	GTCTAAATAGCGAC		Superfamily I DNA and RNA helicases and helicase subunits-related protein
Avin_30380	3150289	3150302	66	-	12.2653	2.49E-05	1	GCCACAAGTTCGTC	*ccmH*	Cytochrome C biogenesis protein
Avin_49000	4961202	4961215	148	-	12.2551	2.50E-05	1	GCCGGTAAACTGGC	*anfH*	nitrogenase iron protein
Avin_04470	424288	424301	129	-	12.2041	2.56E-05	1	GTCAGTTGATCGGC	*cooC*	Carbon monoxide dehydrogenae assesory protein, CooC
Avin_25460	2554247	2554260	158	-	12.2041	2.56E-05	1	GTCGGAAAAGTGTT		hypothetical protein
Avin_48870	4947131	4947144	146	+	12.1224	2.66E-05	1	GCCGGAAAATCGGC	*spuA*	glutamine amidotransferase
Avin_48880	4947131	4947144	117	+	12.1224	2.66E-05	1	GCCGGAAAATCGGC		glutamine synthetase
Avin_25210	2527539	2527552	92	-	12.051	2.75E-05	1	GTCGCAAGAGCGGC		acyl-CoA dehydrogenase
Avin_00370	38375	38388	246	+	12.0408	2.76E-05	1	GTCGGAAATCCGGC		transmembrane protein with C-terminal GGDEF motif
Avin_08030	759646	759659	13	-	12.0306	2.78E-05	1	GCCAGAAAAGCGAT	*mpl*	UDP-N-acetylmuramate:L-alanyl-gamma-D-glutamyl-meso-diaminopimelate ligase
Avin_37860	3829822	3829835	298	+	12.0102	2.80E-05	1	GCCAGAAGATCCAC		conserved hypothetical protein
Avin_01770	166897	166910	142	-	11.9286	2.91E-05	1	GTCTGTTGATCGAC		conserved hypothetical protein
Avin_15680	1537867	1537880	146	+	11.9082	2.94E-05	1	GCCTCAAGACCGAC	*edd-1*	6-phosphogluconate dehydratase
Avin_38290	3874472	3874485	210	+	11.898	2.96E-05	1	GCCACAAAAGCGGC		hypothetical protein
Avin_38310	3874472	3874485	110	+	11.898	2.96E-05	1	GCCACAAAAGCGGC	*mvaT*	Transcriptional regulator MvaT
Avin_35460	3619546	3619559	371	-	11.8878	2.97E-05	1	GCCGGAAAAGCGTC		hypothetical protein
Avin_46200	4695867	4695880	146	-	11.8673	3.00E-05	1	GTCACATGATTGTT		conserved hypothetical protein
Avin_03960	377973	377986	275	+	11.8469	3.02E-05	1	GCCGGAAGTTTGTC		conserved hypothetical protein
Avin_60150	1678775	1678788	-27	+	11.8265	3.05E-05	1	GTCGGTAGAGCGGC	*tRNA*	tRNA-Thr
Avin_24310	2424232	2424245	25	-	11.8265	3.05E-05	1	GCCGCATGATCGAC	*flhC*	Flagellar transcriptional activator
Avin_27020	2771872	2771885	189	+	11.8265	3.05E-05	1	GCCGGATGATCGAC		hypothetical protein
Avin_38420	3887445	3887458	29	-	11.8265	3.05E-05	1	GCCGGTAGAGTGTC		GGDEF Response Regulator
Avin_38440	3887445	3887458	150	-	11.8265	3.05E-05	1	GCCGGTAGAGTGTC	*fumB*	fumarate hydratase, class I
Avin_20420	2034191	2034204	55	-	11.8061	3.08E-05	1	GCCAGTAAAGCGAT	*thrS*	threonyl-tRNA synthetase
Avin_05070	481496	481509	354	-	11.7857	3.10E-05	1	GCCTCAAGAGTGAC	*bcsD*	Cellulose synthase subunit D
Avin_00180	20220	20233	237	-	11.602	3.38E-05	1	GCCGGTTGATCGAC	*lysM*	peptidoglycan-binding LysM protein
Avin_43940	4442339	4442352	243	-	11.5918	3.39E-05	1	GTCGATAATTCCTC		TonB-dependent receptor family
Avin_29140	3006175	3006188	152	-	11.551	3.44E-05	1	GCCAGATGATCGTC		conserved hypothetical protein
Avin_28760	2961921	2961934	176	+	11.4898	3.55E-05	1	GCCGAAAGATCGTT	*nfuA*	NfuA protein
Avin_21990	2198432	2198445	175	-	11.4388	3.63E-05	1	GTCTAAATTTTGTC		ABC transporter, inner membrane permease component
Avin_22000	2198432	2198445	19	-	11.4388	3.63E-05	1	GTCTAAATTTTGTC		transcriptional regulator, Crp/Fnr family
Avin_18540	1833471	1833484	167	-	11.4082	3.68E-05	1	GCCGGAAGATCGGC		outer membrane efflux protein
Avin_20010	1989057	1989070	195	+	11.3673	3.74E-05	1	GTCTCAAAACTGTT	*ccoN*	cytochrome c oxidase, cbb3-type, subunit I
Avin_20020	1989057	1989070	217	+	11.3673	3.74E-05	1	GTCTCAAAACTGTT		conserved hypothetical protein
Avin_05330	513845	513858	-21	+	11.2653	3.90E-05	1	GCCAGAAGATCGTT		Glycosyl transferase, family 2
Avin_40410	4092022	4092035	-6	+	11.2653	3.90E-05	1	GTCGCATGATCGAT	*iscR*	Iron-sulphur cluster assembly transcription factor IscR
Avin_45300	4596506	4596519	85	+	11.1939	4.03E-05	1	GCCTATAATGCGAC	*pilM*	Type IV pilus assembly protein
Avin_45310	4596506	4596519	66	+	11.1939	4.03E-05	1	GCCTATAATGCGAC	*ponA*	Penicillin binding protein 1A
Avin_23100	2306521	2306534	-49	+	11.1837	4.05E-05	1	GCCAGAAGAGCGGC	*oprI*	outer membrane lipoprotein OprI
Avin_34230	3497819	3497832	316	-	11.1327	4.12E-05	1	GCCGGATGATTGTC		conserved hypothetical protein
Avin_14050	1373815	1373828	95	+	11.1224	4.14E-05	1	GTCTCTAGTGCGAC	*bfr*	bacterioferritin
Avin_26730	2743375	2743388	208	+	11.0816	4.22E-05	1	GTCACATATCTCAC		conserved hypothetical protein
Avin_48350	4900880	4900893	62	-	11.0714	4.24E-05	1	GTCGATAATCCCTC		ammonia monooxygenase
Avin_10970	1050741	1050754	101	+	11.0408	4.30E-05	1	GTCAATAAAATGTC	*algD*	GDP-mannose 6-dehydrogenase
Avin_12680	1232969	1232982	14	-	11.0408	4.30E-05	1	GTCTGAAGATCCAC	*maf*	septum formation protein
Avin_40650	4108261	4108274	29	-	11.0204	4.34E-05	1	GTTGAAAATCCGTC		hypothetical protein
Avin_40660	4108261	4108274	384	-	11.0204	4.34E-05	1	GTTGAAAATCCGTC		hypothetical protein
Avin_06280	608611	608624	139	+	11	4.38E-05	1	GTCGCTAATGTCAC	*rplB*	50S ribosomal protein L2

Avin_13260	1289828	1289841	180	-	10.9796	4.40E-05	1	GCCAGATAATCGGC	*murC*	UDP-N-acetylmuramate--alanine ligase
Avin_35200	3594005	3594018	232	+	10.9694	4.42E-05	1	GTCAATAGATTGAA		conserved hypothetical protein
Avin_55010	179363	179376	114	+	10.8878	4.60E-05	1	GTCGATTGTTTCAC	*rRNA*	ribosomal RNA(operon 1/6)
Avin_55040	1379899	1379912	114	+	10.8878	4.60E-05	1	GTCGATTGTTTCAC	*rRNA*	ribosomal RNA(operon 2/6)
Avin_55070	1911170	1911183	114	+	10.8878	4.60E-05	1	GTCGATTGTTTCAC	*rRNA*	ribosomal RNA(operon 3/6)
Avin_55100	2840775	2840788	114	-	10.8878	4.60E-05	1	GTCGATTGTTTCAC	*rRNA*	ribosomal RNA(operon 4/6)
Avin_55130	4279011	4279024	114	-	10.8878	4.60E-05	1	GTCGATTGTTTCAC	*rRNA*	ribosomal RNA(operon 5/6)
Avin_55160	4672946	4672959	114	-	10.8878	4.60E-05	1	GTCGATTGTTTCAC	*rRNA*	ribosomal RNA(operon 6/6)
Avin_05450	523825	523838	285	-	10.8163	4.73E-05	1	GTTGCAAAACTGGC	*pckA*	phosphoenolpyruvate carboxykinase
Avin_29700	3069969	3069982	94	+	10.7959	4.78E-05	1	GCTGGAAGACTGAC		Xanthine/uracil permease family
Avin_50730	5141247	5141260	188	-	10.7857	4.79E-05	1	GTCGATTTTCCGAC	*modA3*	molybdate ABC transporter, periplasmic molybdate-binding protein
Avin_50210	5090337	5090350	126	-	10.7041	4.95E-05	1	GTCGATTGTTCGGC		LamB type porin
Avin_51410	5226642	5226655	20	+	10.7041	4.95E-05	1	GTCGATTGTTCGGC		Glycoside hydrolase, clan GH-D
Avin_05110	490958	490971	100	+	10.6633	5.04E-05	1	GTCGGTTTTTTGAC		hypothetical protein
Avin_05120	490958	490971	349	+	10.6633	5.04E-05	1	GTCGGTTTTTTGAC	*ompR*	two-component response regulator OmpR
Avin_21240	2122413	2122426	54	-	10.6429	5.08E-05	1	GTCGAAAGTTCGGT		hypothetical protein
Avin_40100	4066070	4066083	173	-	10.602	5.17E-05	1	GTCTGAAAATTGGT		hypothetical protein
Avin_40120	4066070	4066083	111	-	10.602	5.17E-05	1	GTCTGAAAATTGGT		2-isopropylmalate synthase
Avin_17200	1704303	1704316	272	+	10.4898	5.40E-05	1	GTCGCATGAGTGGC		CRISPR-associated protein, CT1975
Avin_31670	3277399	3277412	267	-	10.449	5.49E-05	1	GCCAATAATTTCAT	*oprE*	outer membrane porin OprE
Avin_31680	3277399	3277412	121	-	10.449	5.49E-05	1	GCCAATAATTTCAT		ABC transporter, aliphatic sulfonate substrate-binding protein
Avin_02830	268816	268829	345	-	10.4388	5.51E-05	1	GCCAGAAGATCCTC	*gmk*	guanylate kinase
Avin_46860	4755432	4755445	230	+	10.4388	5.51E-05	1	GCCAGAAGATCCTC	*glpE*	Thiosulfate sulfurtransferase
Avin_25090	2511879	2511892	280	-	10.4082	5.57E-05	1	GTCGAAAGTGCGTT		Major facilitator superfamily transporter
Avin_38800	3923961	3923974	337	+	10.3776	5.66E-05	1	GTCGGAAGACTCGC	*kdsA*	3-deoxy-8-phosphooctulonate synthase
Avin_28700	2957096	2957109	298	-	10.3469	5.73E-05	1	GTCGGAAAACCCAT		hypothetical protein
Avin_31060	3209593	3209606	201	-	10.3061	5.83E-05	1	GCCGGATGAGCGAC		hypothetical protein
Avin_35760	3648691	3648704	384	+	10.3061	5.83E-05	1	GCCACAAATGCGAT		Bacterial regulatory protein, LysR family
Avin_11650	1121008	1121021	364	-	10.2653	5.91E-05	1	GCCAGATATTTGAT	*pepA*	Leucyl aminopeptidase
Avin_40980	4135416	4135429	203	+	10.2551	5.93E-05	1	GCCAAATATCCGGC	*cobW*	Cobalamin synthesis protein
Avin_40990	4135416	4135429	-20	+	10.2551	5.93E-05	1	GCCAAATATCCGGC		hypothetical protein
Avin_36530	3715697	3715710	51	+	10.2449	5.96E-05	1	GCCAAAATTTCGGC		hypothetical protein
Avin_36540	3715697	3715710	194	+	10.2449	5.96E-05	1	GCCAAAATTTCGGC		Fic (filamentation induced by cAMP) protein
Avin_16280	1612680	1612693	34	-	10.1939	6.09E-05	1	GTTGATAGTTTGGC		Prevent-host-death protein
Avin_16290	1612680	1612693	240	-	10.1939	6.09E-05	1	GTTGATAGTTTGGC		addiction module toxin, Txe/YoeB family
Avin_08610	818228	818241	233	+	10.1633	6.17E-05	1	GCTACAAGATCGTC	*benC*	Benzoate 1,2-Dioxygenase Reductase
Avin_44610	4514066	4514079	87	+	10.0816	6.38E-05	1	GGCAAAAGACCGAC		3-deoxy-D-manno-octulosonic-acid transferase
Avin_44620	4514066	4514079	144	+	10.0816	6.38E-05	1	GGCAAAAGACCGAC		FAD dependent oxidoreductase
Avin_34150	3489170	3489183	-8	-	10.0612	6.43E-05	1	GTCACATGTGTGGC	*purF*	Amidophosphoribosyl transferase
Avin_01760	165769	165782	175	-	10.0408	6.47E-05	1	GCCAGTTGATCGGC		Thioesterase superfamily protein
Avin_44140	4468473	4468486	294	-	9.9898	6.60E-05	1	GCCTGATGATCGAC		PupR/FecR-like anti-sigma factor protein
Avin_31960	3297143	3297156	-17	+	9.96939	6.66E-05	1	GTCGAAAAACTTAC		transcriptional regulator protein
Avin_45290	4595316	4595329	-22	+	9.95918	6.69E-05	1	GGCAGAAGATTGAC	*pilN*	Fimbrial assembly protein
Avin_32050	3304564	3304577	214	-	9.94898	6.72E-05	1	GTCGCTTGTCCGTC		Conserved hypothetical protein
Avin_32060	3304564	3304577	84	-	9.94898	6.72E-05	1	GTCGCTTGTCCGTC		hypothetical protein
Avin_49000	4961383	4961396	329	-	9.94898	6.72E-05	1	GTCGGTAAATTCTT	*anfH*	nitrogenase iron protein
Avin_43060	4345553	4345566	216	-	9.90816	6.83E-05	1	GTTGATAAATTGGT		GntR family transcriptional regulator
Avin_43070	4345553	4345566	126	-	9.90816	6.83E-05	1	GTTGATAAATTGGT	*lctP*	L-lactate permease
Avin_16660	1650519	1650532	42	+	9.88776	6.89E-05	1	GTCATTAAATTGAG		ISRSO17-transposase protein
Avin_22400	2241291	2241304	47	+	9.88776	6.89E-05	1	GCCGGAAGAGCGGC	*dszA*	xenobiotic compound monooxygenase, DszA family, A subunit protein
Avin_22410	2241291	2241304	19	+	9.88776	6.89E-05	1	GCCGGAAGAGCGGC		hypothetical protein
Avin_22420	2241291	2241304	354	+	9.88776	6.89E-05	1	GCCGGAAGAGCGGC		ABC nitrate/sulfonate/bicarbonate family transporter protein
Avin_32020	3300906	3300919	54	+	9.88776	6.89E-05	1	GTCATTAAATTGAG		conserved hypothetical protein
Avin_32030	3300906	3300919	42	+	9.88776	6.89E-05	1	GTCATTAAATTGAG		ISRSO17-transposase protein
Avin_50370	5107699	5107712	112	-	9.88776	6.89E-05	1	GTCGGATATGCGTC		Aspartate racemase
Avin_13440	1310352	1310365	169	-	9.83673	7.02E-05	1	GCCGAATATCTGGC	*pdxH*	pyridoxamine 5-phosphate oxidase
Avin_20370	2027678	2027691	360	+	9.82653	7.04E-05	1	GCCAGATGTTCGTC		hypothetical protein
Avin_36530	3715697	3715710	51	-	9.82653	7.04E-05	1	GCCGAAATTTTGGC		hypothetical protein
Avin_36540	3715697	3715710	194	-	9.82653	7.04E-05	1	GCCGAAATTTTGGC		Fic (filamentation induced by cAMP) protein
Avin_21480	2148135	2148148	-28	+	9.81633	7.06E-05	1	GTCGGTAAAGCGGT		conserved hypothetical protein
Avin_06770	642530	642543	263	+	9.79592	7.13E-05	1	GTCAAAAGACCGAG	*thiL*	thiamine-monophosphate kinase
Avin_25900	2647458	2647471	309	-	9.77551	7.18E-05	1	GCCAGTAAATCCAT		conserved hypothetical protein
Avin_12400	1205516	1205529	22	-	9.76531	7.20E-05	1	GCCGGAATTCCGAC		PepSY-associated TM helix
Avin_01370	136470	136483	165	-	9.7449	7.27E-05	1	GTGACAAAACTGAC		conserved hypothetical protein
Avin_01380	136470	136483	276	-	9.7449	7.27E-05	1	GTGACAAAACTGAC	*nifH*	Nitrogenase iron protein
Avin_50680	5137060	5137073	126	-	9.7449	7.27E-05	1	GTCGTAAAAATGAC	*modE*	Mo regulation, Mo processing homeostasis
Avin_50690	5137060	5137073	67	-	9.7449	7.27E-05	1	GTCGTAAAAATGAC	*modG*	Mo processing, homeostasis
Avin_22180	2217765	2217778	71	-	9.72449	7.34E-05	1	GTCGATAGACTGCC		hypothetical protein
Avin_22190	2217765	2217778	52	-	9.72449	7.34E-05	1	GTCGATAGACTGCC	*pykA-2*	Pyruvate kinase
Avin_34320	3506487	3506500	272	-	9.72449	7.34E-05	1	GTCACGAGATCGAC	*dusC*	tRNA-dihydrouridine synthase C
Avin_48970	4958268	4958281	59	+	9.72449	7.34E-05	1	GTCAGATAATCCAT	*anfK*	Fe-only nitrogenase, beta subunit
Avin_16360	1618707	1618720	385	-	9.68367	7.44E-05	1	GCTAAATAATTGAT		major facilitator family transporter
Avin_13260	1289828	1289841	180	+	9.61224	7.66E-05	1	GCCGATTATCTGGC	*murC*	UDP-N-acetylmuramate--alanine ligase
Avin_06960	661661	661674	-28	-	9.59184	7.71E-05	1	GTCAAGAAACTGAT	*accB*	acetyl-CoA carboxylase, biotin carboxyl carrier protein
Avin_02470	237126	237139	-20	-	9.58163	7.73E-05	1	GTCGCAAGTTCCTC	*aglA-2*	Alpha-glucosidase
Avin_41820	4218471	4218484	-13	+	9.55102	7.83E-05	1	GTCACAATACTCAT		Lysine 2,3-aminomutase
Avin_41840	4218471	4218484	129	+	9.55102	7.83E-05	1	GTCACAATACTCAT		Bacterial regulatory protein, LysR family
Avin_03160	295073	295086	-1	-	9.5102	7.96E-05	1	GCCGAAAGATCCAT		TonB protein
Avin_43160	4359155	4359168	23	+	9.5102	7.96E-05	1	GCCAGTATTTTGAT		Resolvase-like protein
Avin_13800	1341060	1341073	-25	+	9.47959	8.06E-05	1	GCCGAATATTCGGC		hypothetical protein
Avin_13800	1341060	1341073	-25	-	9.47959	8.06E-05	1	GCCGAATATTCGGC		hypothetical protein
Avin_25390	2546821	2546834	21	+	9.46939	8.09E-05	1	GCCTGATGACCGAC	*fpvI*	RNA polymerase sigma factor, FecI family
Avin_25400	2546821	2546834	136	+	9.46939	8.09E-05	1	GCCTGATGACCGAC	*fpvR*	Anti-sigma factor, FecR family
Avin_26970	2764833	2764846	260	-	9.46939	8.09E-05	1	GTCTTTATTGTGAC		conserved hypothetical protein-transmembrane prediction
Avin_13600	1326076	1326089	245	-	9.45918	8.12E-05	1	GCCACAAGTGCGGC		transposase
Avin_13610	1326076	1326089	64	-	9.45918	8.12E-05	1	GCCACAAGTGCGGC	*ung*	uracil-DNA glycosylase
Avin_45270	4594080	4594093	15	+	9.42857	8.23E-05	1	GTCGTTATAGCGAT	*pilP*	Pilus assembly protein
Avin_48670	4929553	4929566	130	+	9.40816	8.27E-05	1	GTCACAAAGCCGAC		conserved hypothetical protein
Avin_48680	4929553	4929566	117	+	9.40816	8.27E-05	1	GTCACAAAGCCGAC		mcbC-like oxidoreductase
Avin_16480	1633994	1634007	135	-	9.38776	8.36E-05	1	GTCAAAAAAGCAAC		ROK-related protein
Avin_16280	1612585	1612598	129	-	9.35714	8.45E-05	1	GCCGCTTGTCCGAC		Prevent-host-death protein
Avin_16290	1612585	1612598	335	-	9.35714	8.45E-05	1	GCCGCTTGTCCGAC		addiction module toxin, Txe/YoeB family
Avin_23130	2308651	2308664	213	+	9.35714	8.45E-05	1	GCTGATAAAGCGTC	*aroG*	Phospho-2-dehydro-3-deoxyheptonate aldolase, subtype 1
Avin_23140	2308651	2308664	86	+	9.35714	8.45E-05	1	GCTGATAAAGCGTC	*cysB*	LysR family transcriptional regulator protein
Avin_33500	3425509	3425522	-23	+	9.35714	8.45E-05	1	GTCGGAATTGCGTC		esterase, poly(3-hydroxybutyrate) depolymerase
Avin_65010	397467	397480	-45	-	9.30612	8.61E-05	1	GTCGAAGGATCGAC	*ncRNA*	PrrB_RsmZ sRNA family; predicted by Infernal
Avin_65020	400438	400451	-45	-	9.30612	8.61E-05	1	GTCGAAGGATCGAC	*ncRNA*	PrrB_RsmZ sRNA family; predicted by Infernal
Avin_08930	844018	844031	298	-	9.30612	8.61E-05	1	GTCGAAGGATCGAC		conserved hypothetical protein
Avin_08950	844018	844031	188	-	9.30612	8.61E-05	1	GTCGAAGGATCGAC		Staphylococcus nuclease (SNase-like)
Avin_15420	1515400	1515413	-19	-	9.28571	8.68E-05	1	GTCAGTAAATCGAA		Aminotransferase, class I and II
Avin_15430	1515400	1515413	7	-	9.28571	8.68E-05	1	GTCAGTAAATCGAA		hypothetical protein

Avin_15440	1515400	1515413	254	-	9.28571	8.68E-05	1	GTCAGTAAATCGAA		methionine sulfoxide reductase B
Avin_27150	2785625	2785638	237	-	9.28571	8.68E-05	1	GTCAGTAAATCGAA		carboxymuconolactone decarboxylase-like protein
Avin_18470	1828210	1828223	377	+	9.27551	8.73E-05	1	GTTAATAGTGTCAC		histone-like protein
Avin_33210	3395980	3395993	46	+	9.26531	8.76E-05	1	GTCGATTGTTCGTT	*aroF*	phospho-2-dehydro-3-deoxyheptonate aldolase
Avin_33220	3395980	3395993	44	+	9.26531	8.76E-05	1	GTCGATTGTTCGTT		hypothetical protein
Avin_10610	1007035	1007048	192	-	9.2551	8.78E-05	1	GTCTCTATATCGGC	*tonB*	TonB protein
Avin_13440	1310352	1310365	169	+	9.2551	8.78E-05	1	GCCAGATATTCGGC	*pdxH*	pyridoxamine 5-phosphate oxidase
Avin_47760	4845457	4845470	76	+	9.22449	8.91E-05	1	GTTGCAAATCTGAT		Conserved hypothetical protein
Avin_51720	5268477	5268490	116	-	9.22449	8.91E-05	1	GCCAGAAAAGTCGC	*recC*	exodeoxyribonuclease V, gamma subunit
Avin_39540	4003887	4003900	171	-	9.18367	9.03E-05	1	GTCGCTTGTTCGGC	*trmD*	tRNA (guanine-N(1)-)-methyltransferase
Avin_07210	684124	684137	381	+	9.16327	9.10E-05	1	GTCATAAAAGTGCC		hypothetical protein
Avin_07220	684124	684137	118	+	9.16327	9.10E-05	1	GTCATAAAAGTGCC		hypothetical protein
Avin_02800	265147	265160	345	-	9.15306	9.15E-05	1	GCCGCAATATCGGC		Endoribonuclease L-PSP family protein
Avin_32910	3371263	3371276	375	+	9.15306	9.15E-05	1	GCCGATAGATCCGC		Ribonuclease BN protein
Avin_07220	684174	684187	68	+	9.10204	9.30E-05	1	GCCATTATTTTGGC		hypothetical protein
Avin_31780	3287325	3287338	83	+	9.10204	9.30E-05	1	GTCAAAAATTCCGT		hypothetical protein
Avin_31790	3287325	3287338	130	+	9.10204	9.30E-05	1	GTCAAAAATTCCGT		hypothetical protein
Avin_13820	1343015	1343028	265	-	9.08163	9.39E-05	1	GTCACCAGAGTGAC	*recO*	DNA repair protein RecO
Avin_41760	4208971	4208984	257	+	9.08163	9.39E-05	1	GCCAACAGATCGAC	*adh*	2,3-butanediol dehydrogenase
Avin_12400	1205516	1205529	22	+	9.07143	9.43E-05	1	GTCGGAATTCCGGC		PepSY-associated TM helix
Avin_10880	1037836	1037849	110	+	9.06122	9.47E-05	1	GCCGGTATATCGAT	*algV*	Alginate biosynthesis protein AlgV
Avin_13960	1363550	1363563	41	-	9.06122	9.47E-05	1	GCTAATTATCCGAC		Glycosyl transferase, family 51
Avin_12010	1157064	1157077	329	-	8.96939	9.79E-05	1	GCCGGATGATCGGC		conserved hypothetical protein
Avin_12020	1157064	1157077	-37	-	8.96939	9.79E-05	1	GCCGGATGATCGGC		N-acetyltransferase (GNAT) family
Avin_12030	1157064	1157077	385	-	8.96939	9.79E-05	1	GCCGGATGATCGGC		conserved hypothetical protein
Avin_20570	2047912	2047925	160	-	8.94898	9.87E-05	1	GCTGATAAAGTGGC		VacJ-like lipoprotein
Avin_17910	1780597	1780610	305	-	8.93878	9.90E-05	1	GTTGGAAAAGCGGC		Glycosyl transferase, group 1 family protein
Avin_22590	2255867	2255880	109	-	8.93878	9.90E-05	1	GCCGGTAGTCCGGC		Molybdenum-pterin binding domain protein
Avin_52190	5334153	5334166	26	+	8.91837	9.98E-05	1	GCCAGTTTTTCGAC	*atpH*	F1 sector of membrane-bound ATP synthase, delta subunit
Avin_06940	659684	659697	-21	-	8.90816	0.0001	1	GCTGCAAATCCGAC	*prmA*	ribosomal protein L11 methyltransferase

***The start and end coordinates refers to the genome sequence of *A. vinelandii* DJ strain (Genbank accesion: CP001157)**

P. aeruginosa FleQ logo

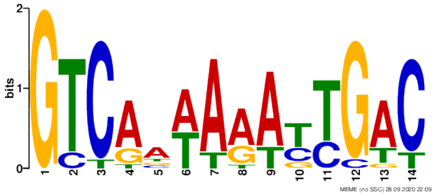

Supplement: Uncited Supplementary Material 1. [file mic-171-01556-s001.pdf]
